# Multidisciplinary care of patients with narcolepsy during coronavirus disease 2019 pandemic in Italy via televisit: the TElemedicine for NARcolepsy feasibility study

**DOI:** 10.1093/sleep/zsac228

**Published:** 2022-09-25

**Authors:** Fabio Pizza, Luca Vignatelli, Claudia Oriolo, Corrado Zenesini, Anastasia Mangiaruga, Andrea Rossetti, Monica Moresco, Stefano Vandi, Francesca Citeroni, Uberto Pagotto, Francesca Ingravallo, Giuseppe Plazzi

**Affiliations:** Department of Biomedical and Neuromotor Sciences (DIBINEM), University of Bologna, Bologna, Italy; UO Epidemiologia e Statistica, IRCCS Istituto delle Scienze Neurologiche di Bologna, Bologna, Italy; UO Epidemiologia e Statistica, IRCCS Istituto delle Scienze Neurologiche di Bologna, Bologna, Italy; Division of Endocrinology and Diabetes Prevention and Care, IRCCS Azienda Ospedaliero-Universitaria di Bologna, Bologna, Italy; Department of Medical and Surgical Sciences (DIMEC), University of Bologna, Bologna, Italy; UO Epidemiologia e Statistica, IRCCS Istituto delle Scienze Neurologiche di Bologna, Bologna, Italy; Department of Medical and Surgical Sciences (DIMEC), University of Bologna, Bologna, Italy; Department of Medical and Surgical Sciences (DIMEC), University of Bologna, Bologna, Italy; UO Epidemiologia e Statistica, IRCCS Istituto delle Scienze Neurologiche di Bologna, Bologna, Italy; Department of Biomedical and Neuromotor Sciences (DIBINEM), University of Bologna, Bologna, Italy; UO Epidemiologia e Statistica, IRCCS Istituto delle Scienze Neurologiche di Bologna, Bologna, Italy; UO Epidemiologia e Statistica, IRCCS Istituto delle Scienze Neurologiche di Bologna, Bologna, Italy; Division of Endocrinology and Diabetes Prevention and Care, IRCCS Azienda Ospedaliero-Universitaria di Bologna, Bologna, Italy; Department of Medical and Surgical Sciences (DIMEC), University of Bologna, Bologna, Italy; Department of Medical and Surgical Sciences (DIMEC), University of Bologna, Bologna, Italy; UO Epidemiologia e Statistica, IRCCS Istituto delle Scienze Neurologiche di Bologna, Bologna, Italy; Department of Biomedical, Metabolic, and Neural Sciences, University of Modena and Reggio Emilia, Modena, Italy

**Keywords:** telemedicine, televisit, management, care, narcolepsy, sleepiness, quality of life

## Abstract

**Study Objectives:**

Narcolepsy is a rare chronic central disorder of hypersomnolence with frequent endocrine-metabolic comorbidities. To address the complex care needs of patients during the COVID-19 emergency, we carried out a feasibility study of the TElemedicine for NARcolepsy (TENAR) protocol with the aim of assessing the feasibility of a multidisciplinary care approach via televisit for patients with narcolepsy.

**Methods:**

A feasibility single open-arm study on the multidisciplinary care of children (>7 y.o.) and adults with narcolepsy who required a follow-up visit was realized during the COVID-19 pandemic emergency period in Italy. The study included a sleep, metabolic, and psychosocial assessment via televisit at baseline, at 6, and at 12 months from the study inclusion period (15th May–26th June 2020).

**Results:**

In total 39 out of 44 eligible patients (89%) entered the study (30 adults, nine children); 37 patients (95%) ended the 12-month follow-up. At baseline, the median Epworth sleepiness scale score (ESS) was 10 (IQR 8–14), and the median body mass index (BMI) was 25.6 (IQR 22.1–30.9). During the follow-up period, the ESS score decreased from the 6th month onward (*p* = 0.003), and BMI decreased at the 1-year follow-up (*p* = 0.047), while there were no differences in depressive and anxiety symptoms, quality of life, compliance with treatment, adverse drug reactions, or accidents.

**Conclusions:**

High response and retention rates, stability of ESS, and lack of side effects indicate that telemedicine is a feasible and safe approach for adults and children with narcolepsy.

## Graphical Abstract

**Graphical Abstract:**
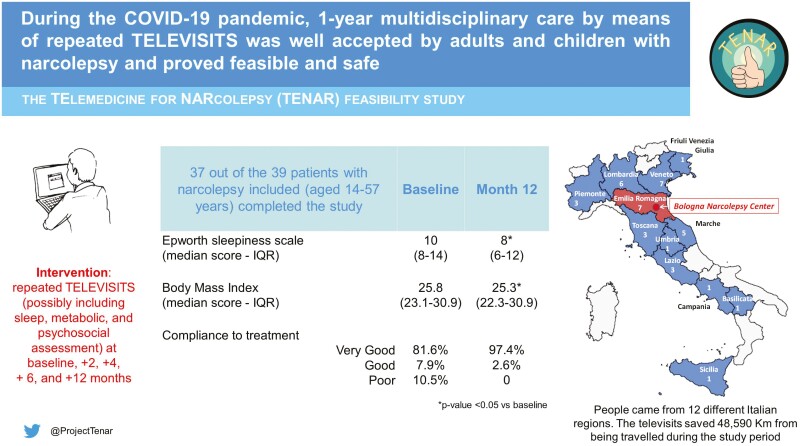

**Clinical Trial Registration**

TElemedicine for NARcolepsy (TENAR). https://clinicaltrials.gov/ct2/show/NCT04316286. Registered on 20 March 2020 with registration number NCT04316286.

**Statement of Significance**

Narcolepsy is a rare disease requiring multidisciplinary care, a need scarcely addressed given the low number of expert centers. During the COVID-19 pandemic emergency period, multidisciplinary care by means of televisits was well accepted by patients and proved to be feasible and safe. Further studies should compare telemedicine with the usual in-person care and expand its application to other rare diseases.

## Introduction

Narcolepsy is a rare chronic central disorder of hypersomnolence [1] with excessive daytime sleepiness (EDS) as the main disabling symptom. Narcolepsy onset occurs between childhood and young adulthood [2]. Metabolic (obesity, type 2 diabetes, and precocious puberty) and psychiatric (depression, anxiety, and eating disorders) comorbidities are additional common features [3], and call for a multidisciplinary approach. Narcolepsy is often diagnosed more than a decade after disease onset [2, 4]. Sleep centers with adequate skills and experience to manage disease complexity [5] are sparse at a national level, thus forcing patients (and their families) to undergo long and expensive journeys, further increasing the disease-related burden [6].

The Narcolepsy Center (CN) of the *IRCCS Istituto delle Scienze Neurologiche di Bologna*, located in the North–East of Italy (Emilia Romagna Region), provides a multidisciplinary management of narcolepsy addressing metabolic, neurodevelopmental, psychiatric, and psychosocial aspects. Follow-up visits are scheduled every 3–6 or 12 months, according to patients’ requirements. In 2018 the CN launched the TENAR (TElemedicine for NARcolepsy) project (granted by Italian Ministry of Health, project code: RF-2016-02364742) with the aim of assessing the efficacy and safety of the televisit (TV) applied to the care of narcolepsy [6] (ClinicalTrials.gov Identifier: NCT04316286). The TENAR project included the first randomized controlled trial (RCT) testing a telemedicine (TM) approach to narcolepsy.

TM is one of the most promising approaches as a means of advancing patient health by improving access to the expertise of qualified sleep medicine specialists [7]. TM is the “practice of medicine using electronic communications, information technology, or other means between a licensee in one location and a patient in another location” [8]. TM includes TVs via videoconference, a synchronous live interactive visit in which “patients and providers are separated by distance but interact in real-time utilizing videoconferencing as the core technology” [7]. TM request and offer has progressively grown in the past decade in the field of sleep medicine, and a growing body of evidence shows that it is effective in the diagnosis and management of obstructive sleep apnea (namely by improving adherence to positive airway pressure therapy) and in the management of insomnia with cognitive behavioral therapy [9]. But it was the COVID-19 pandemic, that drastically curtailed nonemergent health care delivery, that advocated a widespread use of TM to provide health care safely for patients, providers, and staff [10–14].

The scheduled period for the initiation of the TENAR tele-multidisciplinary care RCT coincided with the first epidemic peak of Coronavirus Disease 2019 (COVID-19) in Italy, with the withdrawal of all nonurgent outpatient visits, replaced by telephone consultations. While performing consultations by phone in April–May 2020 [15], we realized that there were patients who needed a more structured contact than a telephone call, usually due to metabolic comorbidities or psychosocial needs. To provide sufficiently sound evidence regarding the care provided by TM during an emergency period, a single open arm pilot study of the TENAR project was devised, aimed at assessing the feasibility of a multidisciplinary approach via TV for patients with narcolepsy during the COVID-19 pandemic emergency in Italy.

## Methods

This study is reported following the CONSORT 2010 statement extension for randomized pilot and feasibility trials [16], ignoring items that are not applicable as recommended for nonrandomized studies [17].

### Design

One-year monocentric single-arm open trial.

### Pandemic situation, population, and setting

The area of Bologna was hit by three epidemic waves of COVID-19 (with the first approximately from March to May 2020, the second from November 2020 to April 2021, and the third from July to September 2021) [18]. Considering the different risks of infection in the corresponding periods, the national and the local health authorities (i.e. Italian Regions) applied different levels of restriction to citizens’ mobility (from strict lockdown to various travel limitations between cities or regions) and healthcare services (cancelation of all scheduled hospital admissions and in-person outpatient visits, or reduction in the number of appointments).

Eligible subjects for the TENAR feasibility study were children (≥ 7 years old) and adults with an established diagnosis of narcolepsy [1], able to use electronic communication devices, living in areas covered by the internet, with a planned outpatients ­follow-up consultation during the study enrollment period (15th May–26th June 2020), and requiring multidisciplinary care. The study enrollment period ended as soon as in-person visits were once again allowed in Italy, and patients were able to travel across the country. Exclusion criteria were the following: inability to read, write, or use a web-based videoconference platform (including no internet access).

### Procedures and intervention

During the period when in-person visits were curtailed, from May 15th to June 26th, 2020, eligible patients (or parents in the case of children) who had a scheduled follow-up visits were invited to participate in the study. Patients who accepted underwent a baseline TV by three experts who independently carried out a clinical assessment of neurological (F.P.), metabolic (C.O.), and psychosocial aspects (A.M. and A.R.). The neurological assessment included a standardized evaluation of medical comorbidity, narcolepsy symptoms, and pharmacological treatments for narcolepsy (including adherence and side effects) to provide adjustments to treatment if required. The metabolic evaluation included the assessment of anthropometric features (Body Mass Index [BMI]), dietary habits, and energy expenditure in order to provide behavioral lifestyle interventions, including a healthy diet and physical activity. The psychosocial assessment included an evaluation of the impact of the disease (including psychological aspects and quality of life) and the occurrence of accidents during the past six months. Differently from the usual practice, in this study (as in the TENAR RCT project) all patients underwent the endocrine/metabolic assessment and counseling on the psychosocial impact of narcolepsy on the same day of the neurologic/sleep assessment.

Then, a standardized neurological TV follow-up was proposed at +2, +4, + 6, and +12 months (end of follow-up) to allow a closer follow-up during the pandemic, while a metabolic and psychosocial TV follow-up was proposed at month +6, and between these periods as well as at month +12, according to patients’ needs. The TVs were performed with the aim of assessing the patient’s condition, his/her need of/response to treatments, and possible therapeutic confirmation or new prescriptions, according to usual practice. A formal report of the neurological consultation was sent by mail to the patients/parents.

The TV was conducted through a suitably equipped PC while patients were connected through a personal device (PC, tablet, and smartphone), using any kind of web-based videoconference platform available.

### Outcomes and statistical analysis

The feasibility of the TV was measured by assessing the number of patients who accepted to participate out of those who were eligible and the number of patients who completed the 12-month follow-up TV out of those who were included. Moreover, the technical quality of TV was assessed at follow-up months +2, +4, and +12. Clinical outcomes were measured to characterize clinical trends (EDS, weight, BMI, overall severity, depressive and anxiety symptoms, quality of life, compliance with treatment, adverse drug reactions, and accidents). Outcomes, related measures, and time points for data collection are summarized in Table 1.

**Table 1.** Study outcomes, measures, and timepoints

Outcomes	Measures	Timepoints (months)
*Feasibility*		
Patients who accepted out of eligible patients	Number (%)	Baseline
Number of patients who completed the study out of included patients	Number (%)	12
Technical quality of televisit	Report of system failure	2, 4, 12
*Clinical*		
EDS	ESS [27] (adults)	0, 2, 4, 6, 12
	ESS-CHAD [28] (children)	
Overall severity	NSS [29]	0, 6, 12
	Global Impression Scale [30](physicians)	
Weight and BMI	Self-reported by patient	0, 2, 4, 6, 12
Depressive and anxiety symptoms	BDI [31] (adults)	0, 6, 12
	STAI [32] (adults)	
Quality of life	SF-36 [33] (adults)	0, 2, 4, 6, 12
Compliance to treatment	Clinical consultation	0, 2, 4, 6, 12
Adverse drug reactions	Clinical consultation	0, 2, 4, 6, 12
Accidents/past 6 months	Interview (patients and parents)	0, 6, 12

EDS, Excessive daytime sleepiness; ESS, Epworth Sleepiness Scale; ESS-CHAD, Epworth Sleepiness Scale for Children & Adolescents; NSS, Narcolepsy Severity Scale; BMI, Body Mass Index; BDI, Beck Depression Inventory; STAI, State-Trait Anxiety Inventory; SF-36, Short Form-36 health survey.

Finally, the potential impact of TV was estimated in terms of kms saved due to the journey that it had not been necessary to take from the patient's home to the CN.

In the descriptive analysis, the continuous variables were presented as means and standard deviation (SD) or median, interquartile range (IQR), and range (min–max). The categorical variables were presented as absolute (n) and relative frequency (%). Depending on the variables’ distribution, *t*-test for paired data or Wilcoxon matched-pairs signed-ranks test were used to compare the continuous variables over time. McNemar’s test was used to compare the categorical variables over time. *P*-value < 0.05 was considered significant. Statistical analyses were conducted using Stata SE version 14.2.

### Data entry, coding, security, and storage

Coordination of the project, data management, analysis, and writing of the report was performed by the CN.

All data were entered electronically in an ad hoc designed web-based e-CRF during the TV (clinical assessment), and directly by the patient who filled in the forms before each visit (e-questionnaires) [6]. The e-CRF is designed and validated for data security and storage according to international standards (US FDA: 21 CFR Part 11; EU GMP: Annex 11; ICH/GCP; VICH/GCP; GAMP 5; GDPR). Personal information about participants was processed in accordance with the GDPR and national regulations on data protection.

### Ethical approval and informed consent procedures

The study was conducted in accordance with the Declaration of Helsinki. The Independent Ethics Committee of Area Vasta Emilia Centro (CE-AVEC) approved the TENAR RCT (number 121/2018/SPER/AUSLBO) and the protocol amendment concerning the feasibility study. The feasibility study was initially planned for a 6 months duration, then extended to 12 months for the neurological follow-up (while other specialists were involved on a strictly clinical need basis). A signed informed consent form was obtained by the sleep experts prior to recruitment. Consent for patients <18 y.o. was provided by their parents. Separate assent forms were signed by minors. Both participants and parents/guardians were free to withdraw their consent for participation at any time.

## Results

Patient enrollment was conducted from May 15, 2020 to June 26, 2020, and the last follow-up visit occurred on August 28, 2021; 44 patients were eligible, and 39 (89%) were accepted to participate. In total 3 out of 44 patients (7%) refused due to a lack of confidence with electronic devices, while two (4%) declined due to their preference for an in-office visit at the end of the emergency. The characteristics of patients included at baseline are reported in Table 2. Almost all patients (38/39) had type 1 narcolepsy, confirmed by cerebrospinal hypocretin-1 assay in 34 cases; 27% (10) were obese and 54% (21) had at least one other disease.

**Table 2.** Patients’ characteristics at baseline (*N* = 39)

Baseline characteristics	Categorical variables: N (%) Continue variables: median (IQR) [min–max]
*Sex*	
Female	17 (43.6)
Male	22 (56.4)
Age children–years	16 (14–16) [14–17]
Age adults–years	35 (22–45) [18–57]
*Education*	
Elementary school	2 (5.1)
Secondary school	11 (28.2)
High school	23 (59.0)
University degree	3 (7.7)
Narcolepsy type 1	38 (97.4)
Narcolepsy type 2	1 (2.6)
Age onset–years	15 (9 – 20) [3 – 45]
Age diagnosis–years	18 (12 – 35) [7 – 46]
*Orexin*	
Positive	34 (87.2)
Negative	2 (5.1)
Missing	3 (7.7)
*Cataplexy*	
Yes	34 (87.2)
No	5 (12.8)
*Cataplexy frequency*	
< 1/year	2 (5.9)
1/year–1/month	8 (23.5)
1/month–1/week	8 (23.5)
1/week–1/day	10 (29.4)
>1/day	6 (17.7)
*Therapy*	
Sodium Oxybate	32 (82.1)
Modafinil	13 (33.3)
Pitolisant	13 (33.3)
Venlafaxine	3 (7.7)
Other	3 (7.7)
*Other diseases*	
Hypertension	5 (13.2)
Restless Legs Syndrome	3 (7.7)
REM sleep behavior disorder	2 (5.1)
Obstructive sleep apnea syndrome	2 (5.1)
Chronic lung diseases	3 (7.7)
Depression	2 (5.1)
Psychosis	1 (2.6)
Hypothyroidism	1 (2.6)
Rheumatoid arthritis	1 (2.6)
Cancer	1 (2.6)

### Feasibility outcomes

**Figure 1.**
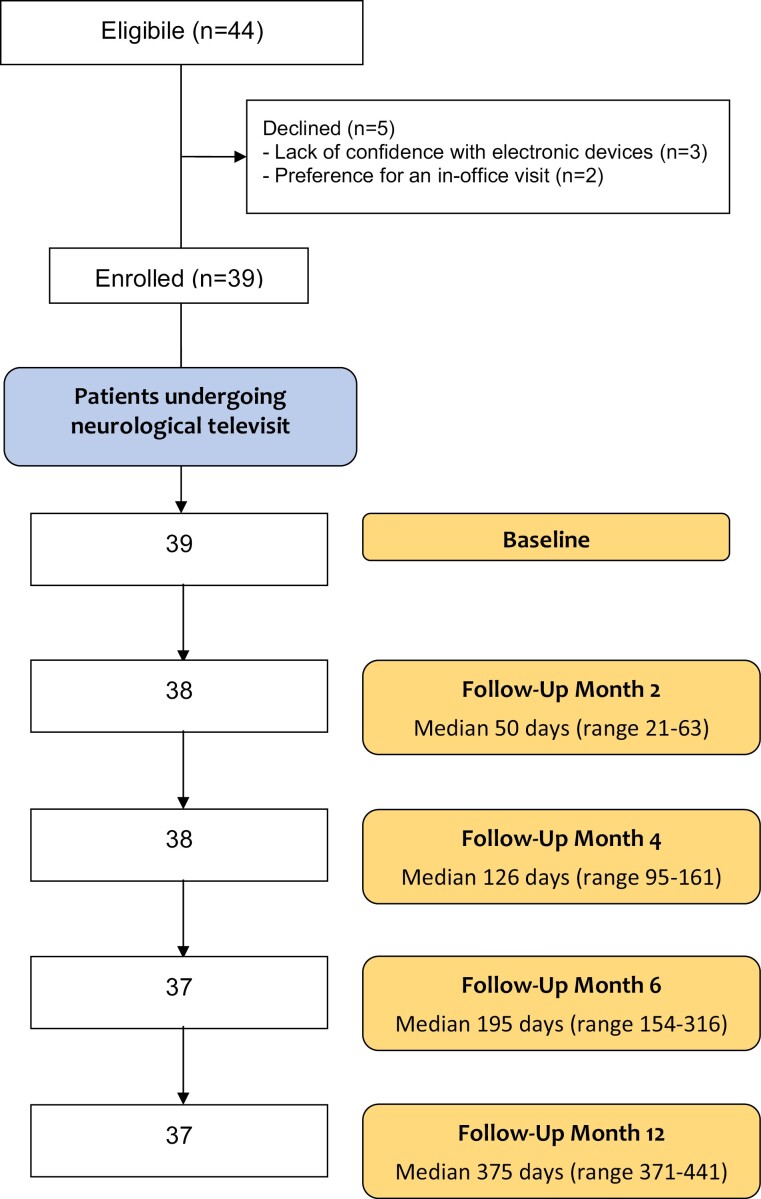
Flowchart of the study. The total number of neurological televisits was 190.

Ninety-five percent of included patients (37 out of 39) completed the study. As detailed in Figure 1, 37 out of 39 patients underwent neurological follow-up TV at both 6 and 12 months. The metabolic assessment was completed by 37 out of 39 patients at month 6 and led to the need for a 12-month follow-up in 11 patients with significant endocrinological comorbidity and needs.

Technical problems and patients’ satisfaction with the TV procedures were assessed at months +2, +4, and +12 as reported in Table 3. Technical problems were more common at the beginning of the project (month 2) and vanished during the 12-month ­follow-up. Overall technical quality of the TV was considered satisfactory by most patients (above 94%) at all time points.

**Table 3.** Technical problems and patients’ satisfaction with the televisit procedures

Technical problem N (%)	Month 2	Month 4	Month 12
	(patients *N* = 38)	(patients *N* = 38)	(patients *N* = 36)
Connection problem	8 (21.1)	2 (5.3)	5 (13.9)
Utilization not easy	2 (5.3)	0 (0)	0 (0)
Televisit interruption	8 (21.1)	2 (5.3)	0 (0)
Televisit not completed	1 (2.6)	0 (0)	0 (0)
Overall technical quality of the televisit not satisfactory	2 (5.1)	1 (2.6)	1 (2.8)

### Clinical outcomes

Clinical outcomes at baseline and at the 12-month follow-up are reported in Table 4. All measures were stable during the period, but EDS (2 points fewer in terms of the median Epworth Sleepiness Scale, ESS, score, *p* = 0.003), weight, and BMI (0.5 points fewer and *p* = 0.047) showed a statistically significant favorable change. Conversely, other measures of disease severity (narcolepsy severity scale and global impression scale), psychosocial impact (depression and anxiety symptoms and quality of life), and compliance/adverse reactions to treatment were stable over time (mood and quality of life measures of the nine adolescents were not reported/contrasted given the small group number). Overall, the occurrence of accidents during the previous 6 months was low at baseline (1 out of 39 patients) and equal to zero at the 12-month follow-up.

**Table 4.** Clinical outcomes at baseline and at 12-month follow-up.

Outcome	N	Baseline	12 months	*p*-value baseline vs 12 months
		Median (IQR) [min–max] Or N (%)		
ESS/ESS-CHAD	33	10 (8–14)	8 (6–12)	0.003
		[3–20]	[3–20]	
NSS in adults	21	18 (17–27)	18 (12–26)	0.36
		[2–52]	[5–47]	
Global impression scale	37	4 (3–4)	4 (3–4)	0.43
		[2–5]	[1–6]	
Weight–kg	38	78 (67–98)	76 (67–93)	0.049
		[43.5–145]	[42–143]	
BMI–kg/m^2^	37	25.8 (23.1–30.9)	25.3 (22.3–30.9)	0.047
		[16.2–43.8]	[15.6–43.5]	
*BMI classes*	37			0.091
Underweight		2 (5.4)	3 (8.1)	
Normal		11 (29.7)	15 (40.5)	
Overweight		13 (21.6)	8 (21.6)	
Obesity		11 (29.7)	11 (29.7)	
BDI in adults	21	5 (1–8)	3 (1–8)	0.94
		[0–17]	[0–25]	
STAI-trait in adults	21	43 (42–46)	43 (41–47)	0.44
		[35–54]	[35–52]	
STAI-state in adults	21	38 (30–47)	33 (30–42)	0.14
		[25–62]	[23–61]	
SF-36 PCS in adults	15	49.2 (45.8–52.8)	49.3 (43.5–53.0)	0.95
		[36.0–55.0]	[34.2–54.2]	
SF-36 MCS in adults	15	41.2 (39.5–43.3)	41.9 (40.0–48.7)	0.33
		[36.7–50.4]	[36.6–50.3]	
*Compliance to treatment*	38			0.069
Very good		31 (81.6)	37 (97.4)	
Good		3 (7.9)	1 (2.6)	
Poor		4 (10.5)	0	
Adverse drug reactions	38	11 (28.9)	4 (10.5)	0.08
Accidents/past 6 months	37	1 (5.0)	0 (0)	1

ESS, Epworth Sleepiness Scale; ESS-CHAD, Epworth Sleepiness Scale for Children & Adolescents; NSS, Narcolepsy Severity Scale; BMI, Body Mass Index; BDI, Beck Depression Inventory; STAI, State-Trait Anxiety Inventory; SF-36, Short Form-36 health survey; PCS, Physical Component Summary; MCS, Mental Component Summary.

When considering the trend over time of the main clinical variables (Table 5), we observed that ESS showed a progressive decrease that reached statistical significance at months +6 and +12 compared to baseline. Conversely, BMI showed a stable statistically significant decrease at all time points except at month +6. Compliance to treatment was substantially very good/good over time and showed a single statistical difference at month +2 when the percentage of poor compliers dropped from 10% to 0%.

**Table 5.** Clinical outcomes at baseline and at 12-month follow-up

Outcome	Baseline	Month 2	Month 4	Month 6	Month 12
*ESS*					
N of patients	33	24	27	33	33
median (IQR)	10 (8–14)	10 (8–13)	9 (6–12)	8 (7–12)	8 (6–12)
[min–max]	[3–20]	[3–18]	[2–23]	[3–20]	[3–20]
*p*-value vs baseline		0.829	0.196	0.013	0.003
*BMI*					
N of patients	37	37	37	37	37
median (IQR)	25.8 (23.1–30.9)	25.4 (23.1–30.2)	25.4 (22.1–29.7)	24.7 (22.8–29.3)	25.3 (22.3–30.9)
[min–max]	[16.2–43.8]	[16.2–43.5]	[15.8–43.8]	[15.6–42.8]	[15.6–43.5]
*p*-value vs baseline		0.016	0.039	0.217	0.047
*Compliance to treatment N (%)*					
N of patients	38	37	38	38	38
Very good	31 (81.6%)	29 (78.4)	34 (89.5)	35 (92.1)	37 (97.4)
Good	3 (7.9%)	8 (21.6)	4 (10.5)	3 (7.9)	1 (2.6)
Poor	4 (10.5%)	0	0	0	0
*p*-value vs baseline		0.041	0. 123	0.127	0.069

ESS, Epworth Sleepiness Scale; BMI, Body Mass Index.

### Potential impact

**Figure 2.**
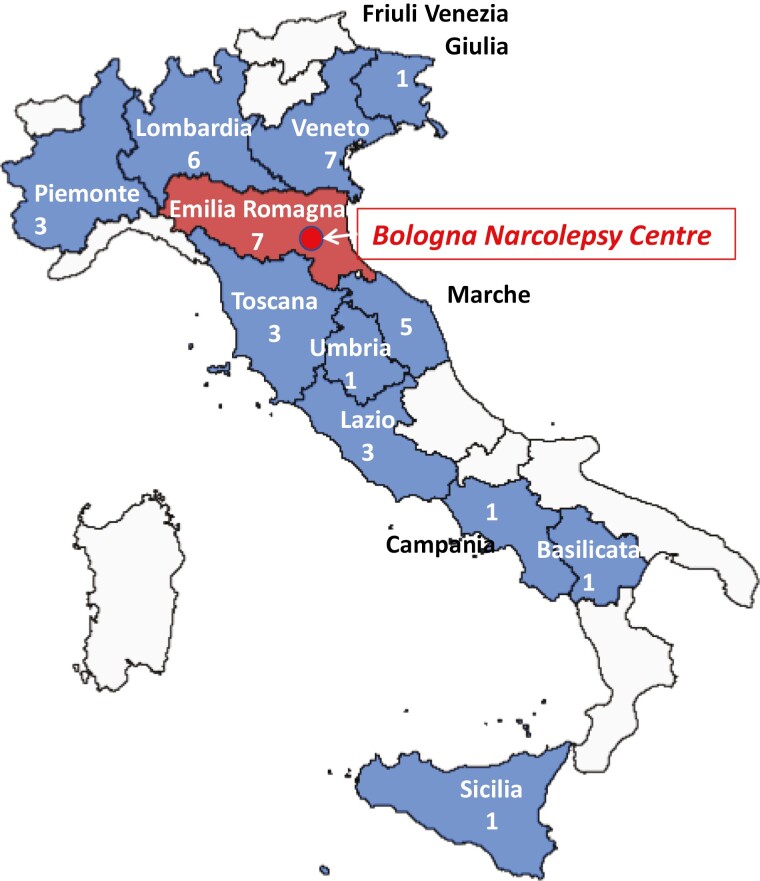
Locations of enrolled patients. Enrolled patients came from 12 different Italian regions. The median distance between Bologna and the patients’ place of residence was 234 km. The application of telemedicine spared an estimated total amount of 48 590 km during the whole study period.

Patients came from 12 different Italian regions (Figure 2); 36 out of 39 (92.3%) were residents outside the city of Bologna. The median distance between Bologna and the patient's city of residence was 234 km (IQR 123–333) [range 48–1221]. The application of the TM approach spared an estimated total amount of 48 590 km during the whole period (9718 km of the journey every 2 months).

## Discussion

Narcolepsy is a rare chronic disease with complex needs encompassing medical and psychosocial aspects. Our study, performed over 1 year in the emergency context due to a pandemic, indicated that TM with TV is feasible and safe in patients with narcolepsy, a disease that requires highly specialized experts and centers: patients’ from all over the country were able to have their needs addressed. Our approach allowed narcolepsy patients to maintain clinical stability despite a dramatic situation linked to the pandemic and the related restrictions that could possibly impact on their wellbeing. Moreover, the application of the TM approach spared an estimated total amount of about 50 000 Km in the whole period, which would have had to be traveled if the visits had been in-person.

In this pilot trial on TM, we reached an overall good level of acceptance on the part of patients with narcolepsy (89%) to participate in the TM follow-up and a substantial adherence to the proposed visits and procedures (only one patient out of 39 did not complete the 1-year follow-up). We are unaware, to date, of similar studies performed in patients with narcolepsy to allow for a direct comparison. Several trials applied TM to patients with obstructive sleep apnea syndrome (OSAS), mainly aimed at improving adherence to continuous positive airway treatment, but showed lower participation rates. Schoch and coworkers were able to randomize only approximately 50% of consecutively attending OSAS patients [19], and reported that both logistical (center-related) and patient-centered factors (e.g. time constraints, unwillingness to participate) prevented higher participation rates, without further details. Other studies did not even detail the rate of acceptance of their TM protocol [20, 21], and briefly mentioned the loss of approximately 5% of the enrolled patients across the study for generical reasons (e.g. withdrawal of consent, unexpected hospitalization, or loss of baseline data) [21]. Our high acceptance rate may be explained by the lack of widespread reference centers for narcolepsy (as for several rare diseases), the alternative option of a simple telephone visit, and the distances between patients’ homes and our center. All these factors, and the clinical impression of a generally high acceptance of patients with narcolepsy for scientific research, may have favored adherence to our TM study. Only a few patients (11%) did not accept the protocol, the main reason being their inability to use electronic devices (7%), an objective obstacle to the possibility of performing any TM approach.

Our clinical approach of short-term repeated follow-up by means of TM was associated with the improvement in EDS and BMI across the pandemic restrictions and up to the 1-year follow-up.

During the pandemic patients with narcolepsy showed a trend toward later bedtimes, suggesting a circadian misalignment, lower adherence to pharmacological treatments, and more prominent EDS [22]. At the same time, when telework was allowed, patients with narcolepsy displayed a tendency to increase the time spent asleep during nighttime with delayed timing for going to bed and morning awakening [23]; they tended to have planned daytime naps more regularly [15], and reported a consequent improvement in EDS [15, 23]. In our study, we did not consider work organization (e.g. telework), and we found that ESS remained substantially stable across the quarantine/restrictions period, with a significant improvement during the 6 months and 1-year follow-up (which occurred in a period that was free from restrictions). We hypothesize that this result was obtained by applying a strict clinical follow-up that was feasible only by means of TM and that was able to maximize adherence to pharmacological treatment, applying subtle modifications of pharmacological and nonpharmacological approaches based on the evolving context of each patient across time.

In parallel, our patients showed an improvement in their BMI, in contrast with previous findings in narcolepsy [15], as well as in the general population [24] during the pandemic emergency in Italy. Various clinical and laboratory data have been collected in order to understand the relationship between food intake, physical activity, and body weight in patients with narcolepsy [25]. We believe that this important result is due to the structured clinical intervention performed in TM by an endocrinologist who was able to prescribe changes in dietary habits and provide indications concerning physical activity when required. In addition, according to the clinical endocrinological criteria alone, about 30% of patients required standardized management for metabolic control over time. This finding highlights the urgent need for multidisciplinary management of narcolepsy, including endocrinological-metabolic expertise, in routine clinical practice.

These results should, however, be considered with caution given the single open-arm study design, the small sample size, and the self-reported nature of the data. In addition, specific questionnaires were used for the nine children included, a number that does not allow statistical comparison within subjects. However, we used tools that are commonly adopted in the field of sleep medicine (ESS), and the neurologist adopted clinical decisions tailored to patients’ needs at each evaluation. The ongoing RCT (TENAR) will include a more structured endocrine/metabolic evaluation, with in-person anthropometric data collection and blood tests at baseline and at the end of the study, and with pharmacological intervention when required. We hope that it will emerge that the TM protocol is not inferior to an in-person multidisciplinary approach to narcolepsy. Finally, since almost all patients had narcolepsy type 1, we cannot generalize the results to include all patients with narcolepsy. However, we can argue that there are no major clinical reasons why patients with narcolepsy type 2 should show particularly different feasibility results

The experiences that all of us had across the prolonged pandemic condition are pushing toward a change in the way we conceive medical care. While sleep medicine practitioners reported that after the pandemic virtual clinic visits are expected to be performed much more frequently [14], this need may be even more urgent in the case of rare diseases like narcolepsy for which the need for centers that are able to provide multidisciplinary patient-centered care is considered by patients, families, and sleep physicians a priority in the case of younger patients [26]. Indeed, only expert centers may provide a qualified multidisciplinary approach by merging different types of expertise that focus on the different aspects of the disease [26]. Our study showed, for the first time, that a TM approach is feasible and safe for children and adults with narcolepsy.

## Data Availability

The study data are available upon request.

## Notes

**Institution where the work was performed:** IRCCS Istituto delle Scienze Neurologiche di Bologna, Bologna, Italy.
